# Laxative Abuse Cessation Leading to Severe Edema

**DOI:** 10.7759/cureus.15847

**Published:** 2021-06-23

**Authors:** Aditya Ragunathan, Pratishtha Singh, Kiranpreet Gosal, Nicolina Scibelli, Victor Collier

**Affiliations:** 1 Internal Medicine, Grand Strand Medical Center, Myrtle Beach, USA

**Keywords:** laxative abuse, edema, adverse drug reaction, simulant laxative, diuretic therapy

## Abstract

Stimulant laxatives are a common class of laxatives that is abused by patients with eating disorders. We present a case of a 30-year-old female who presented with dyspnea, peripheral edema and weight gain who had been chronically using laxatives. Her symptoms were consistent with rebound edema caused by sodium and free water shifts with abrupt cessation of excessive stimulant laxative use. This case highlights the use of furosemide as the mainstay treatment for rebound edema and weight gain.

## Introduction

Stimulant laxatives are the most common class of laxatives abused by patients with eating disorders. While less common than self-induced vomiting, laxative use is the second most common mode of purging noted in patients with bulimia nervosa [1]. Depending on their mechanism of action, laxatives can be grouped into five different classes: bulk, osmotic, surfactants, emollients, and stimulants. Of the various classes used, the one associated with the most medical complications is stimulant laxatives, which not only increase intestinal motor activity but also alter electrolyte transport [1]. The medical complications of laxatives can be split into two main categories, due to their effect on the gastrointestinal system with overall hypovolemia and due to electrolyte abnormalities. The presentation can vary widely however abrupt cessation can lead to pulmonary as well as peripheral edema and weight gain. Treatment focuses on the correction of associated electrolyte abnormalities and symptom improvement.

## Case presentation

We present a case of a 30-year-old female with a past medical history of constipation with chronic laxative use who presented with complaints of peripheral edema and weight gain. The patient had been using stimulant laxatives (sennosides 17.2 mg once to twice a day) for four years but stopped one week prior to presentation due to painless bright-red blood per rectum. The patient denied any other medication use. She initially presented to the emergency department seven days after stopping her laxatives with peripheral edema, progressive shortness of breath, orthopnea, and an 8.62-kg weight gain (weight increased from 56.2 kg to 64.8 kg). Physical exam showed abdominal distention and edema in bilateral lower extremities. On laboratory examination, her potassium was 3.3 mmol/L and N-terminal pro-brain natriuretic peptide (NT-ProBNP) was elevated at 676 pg/mL. Albumin was normal and her urinalysis was negative for protein. She received furosemide 40 mg with improvement in edema and shortness of breath. She was seen two days later at her primary care clinic. At that time, her dyspnea had resolved; however, she had persistent edema and minimal weight loss. Her physical exam showed abdominal distension and pitting edema of the lower extremities. She had no elevated jugular venous pressure, S3 or displaced point of maximal impulse (PMI) on the exam. Her basic metabolic panel was within normal limits and she was started on 40 mg of furosemide daily. An echocardiogram showed a preserved ejection fraction (EF) of 55%-60% with mild mitral and tricuspid regurgitation. The patient denied having prior echocardiograms. She returned to the clinic one week later with resolution of her edema after treatment with furosemide and had returned to her baseline weight. She followed up at the cardiology clinic and no additional medications were added for the patient's treatment.

## Discussion

Stimulant laxatives are the most common class abused by patients with eating disorders [1]. Abuse of stimulant laxatives can lead to several medical complications as these have been shown to act like diuretics at high doses [2]. They can induce sodium and free water loss and abrupt cessation can cause significant rebound edema [2,3]. The systemic effects emanate from hypovolemia and electrolyte disturbances that occur when the body's compensatory mechanisms take over. Electrolytes such as potassium are lost and hypokalemia can further slow down intestinal motility [1]. A hyperaldosterone state can result in long term laxative induced hypovolemia upregulating the renin-angiotensin-aldosterone system. This is due to underlying vascular insensitivity, which reflexively, through a positive feedback mechanism, leads to a state of increased renal retention of sodium, bicarbonate, and water while decreasing serum potassium. A hyperaldosterone state continues despite discontinuation of laxative use leading to rebound edema [4]. This is a self-perpetuating cycle in which hormonal and chemical abnormalities at any point can cause and worsen other abnormalities. Treatment involves loop diuretics. Aldosterone antagonists have also been used in management for patients who meet the criteria for pseudo-Bartter syndrome [2,4-6]. Patients with pseudo-Bartter syndrome present with hypokalemia, metabolic alkalosis, and edema [7]. Hypokalemia is the most dangerous complication of the above mentioned. When a patient presents with electrolyte abnormalities, treatment should focus on repleting electrolyte abnormalities as well as symptom improvement. A loop diuretic was used as our patient had normal potassium and bicarbonate and did not meet the criteria for pseudo-Bartter syndrome. The patient's laxative use should be quantified upon initial encounter and they should be monitored closely for symptom development after cessation. Treatment duration was ill-defined in previous literature; however, it is recommended that treatment should be started at the onset of the first identification of peripheral edema. In our case, treatment persisted until the patient stated symptoms had resolved.

## Conclusions

Abrupt cessation of high dose laxatives can cause edema and rapid weight gain due to fluid shifts and electrolyte disturbances. Our case report highlights a significant amount of weight gain, which has been reported rarely. The optimal treatment approach remains ill-defined at this time but is centered around diuretic use in patients with significant fluid overload.
